# Population Structure and Genetic Diversity of Nile Tilapia (*Oreochromis niloticus*) Strains Cultured in Tanzania

**DOI:** 10.3389/fgene.2019.01269

**Published:** 2019-12-20

**Authors:** Redempta A. Kajungiro, Christos Palaiokostas, Fernando A. Lopes Pinto, Aviti J. Mmochi, Marten Mtolera, Ross D. Houston, Dirk Jan de Koning

**Affiliations:** ^1^Department of Animal Breeding and Genetics, Swedish University of Agricultural Sciences, Uppsala, Sweden; ^2^Department of Aquatic Science and Fisheries, College of Agricultural Sciences and Fisheries Technology, University of Dar es Salaam, Dar es Salaam, Tanzania; ^3^Institute of Marine Sciences, University of Dar es Salaam, Dar es Salaam, Tanzania; ^4^The Roslin Institute and Royal (Dick) School of Veterinary Studies, University of Edinburgh, Edinburgh, United Kingdom

**Keywords:** aquaculture, population structure, genetic diversity, Nile tilapia, double-digest restriction site-associated DNA-sequencing

## Abstract

Understanding population structure and genetic diversity within and between local Nile tilapia lines cultured in Tanzania is important for sustainable aquaculture production. This study investigated the genetic structure and diversity among seven Nile tilapia populations in Tanzania (Karanga, Igunga, Ruhila, Fisheries Education and Training Agency, Tanzania Fisheries Research Institute, Kunduchi, and Lake Victoria). Double-digest restriction site-associated DNA (ddRAD) libraries were prepared from 140 individual fish (20 per population) and sequenced using an Illumina HiSeq 4000 resulting in the identification of 2,180 informative single nucleotide polymorphisms (SNPs). Pairwise F_st_ values revealed strong genetic differentiation between the closely related populations; FETA, Lake Victoria, and Igunga and those from TAFIRI and Karanga with values ranging between 0.45 and 0.55. Population structure was further evaluated using Bayesian model-based clustering (STRUCTURE) and discriminant analysis of principal components (DAPC). Admixture was detected among Karanga, Kunduchi, and Ruhila populations. A cross-validation approach (25% of individual fish from each population was considered of unknown origin) was conducted in order to test the efficiency of the SNP markers to correctly assign individual fish to the population of origin. The cross-validation procedure was repeated 10 times resulting in 77% of the tested individual fish being allocated to the correct population. Overall our results provide a new database of informative SNP markers for both conservation management and aquaculture activities of Nile tilapia strains in Tanzania.

## Introduction

Tanzania is a diversity hotspot of tilapias including more than 30 *Oreochromis* species of which 10 are only found in the country (Genner et al., 2018; Shechonge et al., 2019). *Oreochromis niloticus* is the most widespread tilapiine cichlid both in Tanzania and worldwide. During the last 5 years, Nile tilapia aquaculture in Tanzania has increased from 958 MT in 2011 to 4080 MT in 2017 (Kajungiro et al. unpublished data) with a continuously increasing demand for further expansion. Despite the interest and potential of tilapia aquaculture to contribute to local food production, currently no selective breeding program exists in Tanzania—a situation typical of many African nations.

Common hatchery aquaculture practices could result in a rapid reduction of the genetic diversity of the farmed animals. A well-managed breeding program on the other hand would enable cumulative genetic improvement of target traits, while simultaneously minimize inbreeding and loss of diversity. Forming a base population containing high genetic diversity will be crucial for the success of any future breeding program in Tanzania (Fernández et al., 2014; García-Ballesteros et al., 2017). Furthermore, introductions of fish from one region to another have affected the genetic diversity and population structure of many teleost fish species (Basiita et al., 2018). Due to mismanagement and uncontrolled movement of fish from different regions there is limited information relating to the genetic structure of Nile tilapia strains and their distribution in Tanzania.

Tilapia species have a very complex genetic structure, in common with many other Cichlid fish species (Bezault et al., 2011). Moreover, hybridization and introgression are fairly common in tilapias constituting the management of both wild and farmed populations particularly challenging (Shirak et al., 2009; Wu and Yang, 2012). The aforementioned issue is further exacerbated by the common situation of reproductive viable hybrids in tilapias (Wohlfarth and Hulata, 1982). In addition, ecological factors such as environmental heterogeneity and geographic connectivity have shaped the current population structure and distribution of Nile tilapia in Africa (Bezault et al., 2011).

Genetic diversity plays a crucial role in the adaptation ability of a population in the face of fluctuating environmental conditions (Markert et al., 2010). Conservation programs aim to minimize the loss of genetic diversity in order to increase the chances of successful population restoration and long-term viability. Translocation of fish to supplement suppressed populations may have in fact harmful effects if the recipient population is genetically different (Allendorf and Luikart, 2007). Available knowledge regarding the genetic diversity of cultured strains can also assist in genetic improvement, rearing management and performance potential in various culture environments (Angienda et al., 2011). Further, in selective breeding programs the genetic diversity between and within breeds and populations can provide valuable information regarding the potential response to selection (Oldenbroek, 2017). Due to a high demand from aquaculture, Nile tilapia strains and other unknown tilapia species have been introduced outside their natural geographical distributions in Tanzania (Philippart and Ruwet, 1982; Shechonge et al., 2019). In addition, hybridization with the local tilapia species has been recently reported (Shechonge et al., 2018).

Genetic markers offer a reliable approach for unveiling the genetic structure both among and within populations. In addition, genetic markers can assist in identifying species, individuals or population of origin of unknown samples allowing the authorities in monitoring protected nature reserved areas. As such, knowledge of population genetic structure and genetic diversity of *O. niloticus* is crucial both for conservation practices and for fish breeders. Previous studies examined the genetic structure and diversity between populations of Nile tilapia (*Oreochromis niloticus*), based either on phenotypic traits (Trewavas, 1983), allozymes (Sodsuk and McAndrew, 1991), mitochondrial DNA (Romana-Eguia et al., 2004), randomly amplified polymorphic DNA (Hassanien et al., 2004) or microsatellites (Bhassu et al., 2004; Hassanien and Gilbey, 2005; Mireku et al., 2017). However, the genetic markers used to date have limitations regarding their maximal resolution in detecting the complex genetic structure typically encountered in Nile tilapia populations. Furthermore, to our knowledge no prior study attempted to test the efficiency of genetic markers for predicting the population of origin in putative unknown tilapia samples.

Next-generation sequencing (NGS) technologies have facilitated the discovery of large numbers of genetic markers for practically any organism at an affordable cost allowing the investigation of genetic diversity within and between populations (Candy et al., 2015). Restriction-site associated DNA (RAD) and double-digest RAD (ddRAD) sequencing are NGS-based techniques providing a reduced representation of the studied genome (Baird et al., 2008; Peterson et al., 2012). ddRAD-seq and similar genotyping by sequencing techniques rely on digestion of the genomic DNA with restriction enzyme(s), and subsequent high-depth sequencing of the flanking regions of the cut site. Such genotyping by sequencing techniques have been widely applied in aquaculture species (Robledo et al., 2018). Several studies have applied ddRAD-seq sequencing to generate high-density linkage maps (Brown et al., 2016; Manousaki et al., 2016) and estimate genetic diversity (Antoniou et al., 2017; Hosoya et al., 2018). Furthermore, ddRAD-seq has been utilized in several tilapia studies for evaluating the suitability of DNA from skin mucus swabs (Taslima et al., 2017), identification of sex determining regions (Wessels et al., 2017), and quantitative trait loci (QTL) analysis (Li et al., 2017).

The current study investigated the population genetic structure of seven Nile tilapia populations from Tanzania using ddRAD-seq derived single nucleotide polymorphisms (SNPs). Genetic diversity parameters and population structure using both multivariate analysis and Bayesian clustering algorithms were evaluated. Admixture levels between the different populations were estimated providing valuable information for future management of Nile tilapia resources in Tanzania. Finally, a cross-validation scheme was applied in order to test the efficiency of the generated SNPs for assignment of individual fish to their population of origin.

## Materials and Methods

### Ethics Statement

This study was carried out in accordance with the law on the protection of animals against cruelty (Act no. 12/1974. of the United Republic of Tanzania) upon its approval by the department of Zoology and Wildlife Conservation, University of Dar es salaam. All the permits required to sample wild animals in Tanzania were adhered; these include Research clearance from Tanzania Commission for Science and Technology (COSTECH) and other relevant authorities.

### Fish Sample Collection and Preparation

**Figure 1:**
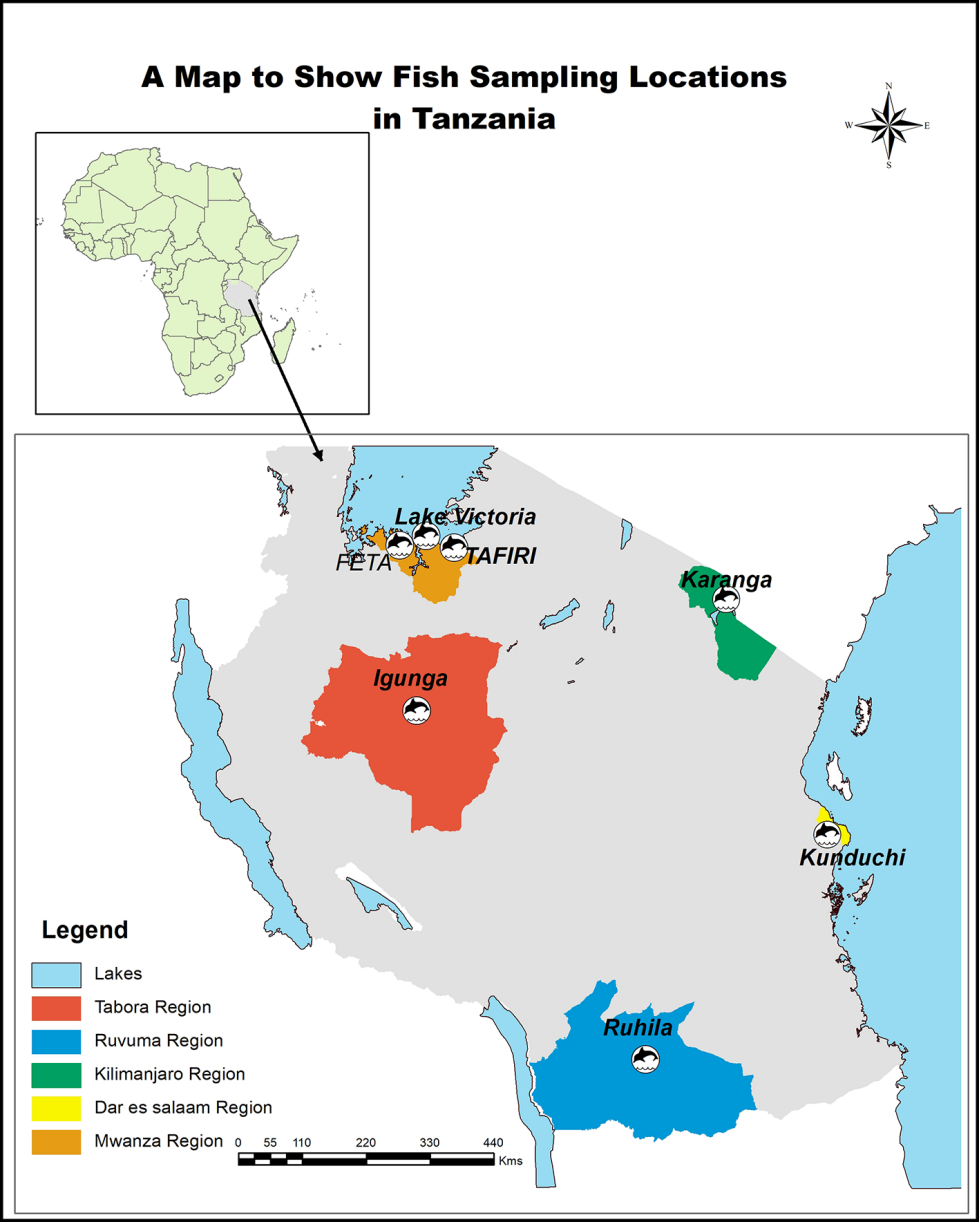
Sampling locations of fish used in the present study.

Farmed stocks of *Oreochromis niloticus* juveniles were collected in 2017 from Government aquaculture centers distributed throughout Tanzania. In particular we collected animals from six farmed populations namely: Tanzania Fisheries Research Institute (TAFIRI; −2.5805° S, 32.8979° E), Fisheries Education and Training Agency (FETA; −2.5851° S, 32.8980° E), Karanga (−3.373680° S, 37.318390° E), Igunga (−4.285810° S, 33.879020° E), Kunduchi (−6.670220° S, 39.214840° E), Ruhila (−10.665510° S, 35.645040° E, and one natural population from Lake Victoria (−2.556348° S, 32.881061° E) (**Figure 1**). FETA and TAFIRI are located along Lake Victoria. The TAFIRI stock originated from Lake Victoria in 2014, while the other populations (FETA and Igunga) were stocked in 2016 (personal communication with fish farmer). Igunga is located in the central part of the country, Karanga in the northern part, Kunduchi along the coast of the Indian Ocean, and Ruhila in the southern part of Tanzania (**Figure 1**). Fish were kept in separate hapas (2 m × 2 m) within an earthen pond at Kunduchi Campus for 4 months. Species identification was based on both prior available records for each population and on morphology characteristics as explained by Trewavas (1983): In particular *O. niloticus* were distinguished from other species by large deep-bodied size with relatively small heads and the presence of regular vertical stripes throughout the depth of caudal fin. A total of 140 fish weighing from 50 to 150 g were used in the study. The fish were sedated using pure clove oil at the dosage of 2 ml clove oil to 20 L of water (Fernandes et al., 2017). Twenty fish from each population were fin clipped. Fin clips were stored in 95% ethanol at −20°C, until DNA extraction.

### DNA Extraction

Genomic DNA was extracted from 0.02 g of fish fin using a spin column (QIAsymphony DSP DNA Mini Kit; Qiagen, Hilden, Germany) and eluted into 100 µl of AE (EDTA) buffer (Qiagen) according to the manufacturer’s tissue protocol and procedures. The purity and concentration of the extracted DNA were quantified using Qubit 2.0 Fluorometer (Invitrogen). Samples were diluted with Tris EDTA (TE) buffer (Thermo Fisher Scientific) to 25 ng/µl and 2 µl were run on a 1% agarose gel by electrophoresis. Diluted samples were stored at −20°C.

### Double-Digest Restriction Site-Associated DNA Library Preparation and Sequencing

ddRAD library preparation was performed according to Peterson et al. (2012), with minor modifications described in Palaiokostas et al. (2015). Briefly, each sample (25 ng DNA) was digested at 37°C for 60 min with *Sbf*I (recognizing the CCTGCA|GG motif) and *Sph*I (recognizing the GCATG|C motif) high fidelity restriction enzymes (New England Biolabs, UK; NEB), using 6 U of each enzyme per microgram of genomic DNA in 1× Reaction Buffer 4 (NEB). The reactions (5 µl final volumes) were then heat inactivated at 65°C for 20 min. Individual-specific combinations of P1 and P2 adapters, each with a unique 5 or 7 bp barcode, were ligated to the digested DNA at 22°C for 60 min by adding 1 µl *Sbf*I compatible P1 adapter (25 nM), 0.7 µl *Sph*I compatible P2 adapter (100 nM), 0.06 µl 100 mmol/L rATP (Promega, UK), 0.95 µl 1× Reaction Buffer 2 (NEB), 0.05 µl T4 ligase (NEB, 2 × 10^6^ U/ml) and reaction volumes made up to 8 µl with nuclease-free water for each sample. Following heat inactivation at 65°C for 20 min, the ligation reactions were slowly cooled to room temperature (over 1 h) then combined in a single pool (for one sequencing lane) and purified. Size selection (300–600 bp) was performed by agarose gel separation and followed by gel purification and PCR amplification. A total of 100 µl of the amplified libraries (13–14 PCR cycles) was purified using an equal volume of AMPure beads. After eluting into 20 µl EB buffer (MinElute Gel Purification Kit, Qiagen, UK), the libraries were ready for sequencing. The libraries were sequenced at Edinburgh Genomics Facility, University of Edinburgh on an Illumina HiSeq 4000 instrument.

### Sequence Data Analysis and Single Nucleotide Polymorphism Genotyping

Reads of low quality (Q < 20) and missing the expected restriction sites were discarded. The retained reads were aligned to the *O. niloticus* reference genome assembly [Genbank accession number GCA_001858045.2 (Conte et al., 2017)] using bowtie2 (Langmead and Salzberg, 2012). Stacks v2 (Catchen et al., 2011; Rochette et al., 2019) was used to identify and extract single nucleotide polymorphisms (SNPs) using gstacks (settings: –*var-alpha 0.001* –*gt-alpha 0.001* –*min*-*mapq 40*). Stacks v2 primarily identified ddRAD loci corresponding to restriction enzyme cutting sites using a sliding window strategy (1 Kbp in length) in the sets of aligned reads on each sample iteratively. Upon data acquisition from all samples on each tested locus, the window was advanced to the next read beyond the previous window bound (Rochette et al., 2019). SNP calling was performed using a Bayesian genotype caller (BGC) allowing a per-nucleotide sequencing error (Maruki and Lynch, 2017). During variant calling, for numerical stabilization reasons a sequencing error under the assumption of polymorphism of at least 0.1 was assumed and the obtained genotype likelihoods were rescaled in order to be greater or equal to 1 (Rochette et al., 2019). Only one single SNP from each individual ddRAD locus was considered for downstream analysis in order to minimize the possibility of genotypic errors and reduce computational time. SNPs with a minor allele frequency (MAF) < 0.05 within a population were discarded. Finally, only SNPs that were detected in at least 75% of the samples in each population were retained for downstream analysis. The aligned reads in the format of bam files were deposited in the National Centre for Biotechnology Information (NCBI) repository under project ID PRJNA518067. The accession numbers of samples analyzed in this study are given in **File S1**.

### Genetic Similarity and Relationship Among Populations

Mean observed (Ho) and expected (He) heterozygosity and average individual inbreeding coefficients (Fis) were estimated using Stacks v2 (Rochette et al., 2019). The R package StAMPP (Pembleton et al., 2013) was used to perform an Analysis of Molecular Variance (AMOVA) using 100 permutations. Additionally, pairwise F_st_ values were obtained using the stamppF_st_ function according to Cockerham and Weir (1984). Furthermore, confidence intervals and p-values of the pairwise F_st_ values testing for significant deviations from zero were estimated using 1,000 bootstraps. Principal component analysis (PCA) was carried out using the R package ADEGENET version 2.1.1 (Jombart et al., 2018).

### Genetic Structure and Admixture

In this study, discriminant analysis of principal components (DAPC) and Bayesian-model-based approaches were used to infer the genetic structure of *O. niloticus* samples derived from 7 populations in Tanzania. Population structure and potential admixture between the different populations was evaluated using Bayesian clustering approaches implemented in the program Structure v2.3.4 (Pritchard et al., 2000). The number of clusters tested (K) ranged from 1 to 9. Markov chain Monte Carlo of 200,000 iterations with a burn-in period of 100,000 were carried for each K-value. The delta-K method based on the criteria proposed by Evanno et al. (2005) and the obtained posterior probability values (Pritchard et al., 2000) were used to determine the optimal number of clusters. Structure results were interpreted using Structure Harvester (Earl, 2012) and CLUMPAK (Kopelman et al., 2015) for identifying the most probable number of clusters. Population structure was further confirmed using DAPC as demonstrated by Jombart et al., (2010). DAPC transformed the data using a prior PCA step and subsequently applied a discriminant analysis step (Jombart and Collins, 2015). The Bayesian Information Criterion (BIC) was used for selecting the optimal number of clusters (K) based on the elbow method (Jombart et al., 2010).

### Population Assignment and Diagnostic Single Nucleotide Polymorphisms

A four-fold cross-validation scheme was applied using the R package ADEGENET version 2.1.1 (Jombart et al., 2018) in order to test the efficiency of the SNP dataset for correctly identifying the population of origin of putatively unknown tilapia samples. The population of origin of 25% of individual fish from each genotyped population (five animals per population) was masked and was used as a test dataset. Predictions regarding population of origin on the aforementioned test set were performed using information obtained through DAPC (*predict.dapc*) on the remaining training data set. The entire procedure was repeated 10 times in order to minimize potential bias due to sample allocation in the training/test datasets. Furthermore, DAPC carried out on the entire dataset was used to identify SNPs with highest population discriminatory value.

## Results

### Double-Digest Restriction Site-Associated DNA Sequencing and Single Nucleotide Polymorphism Identification

**Figure 2:**
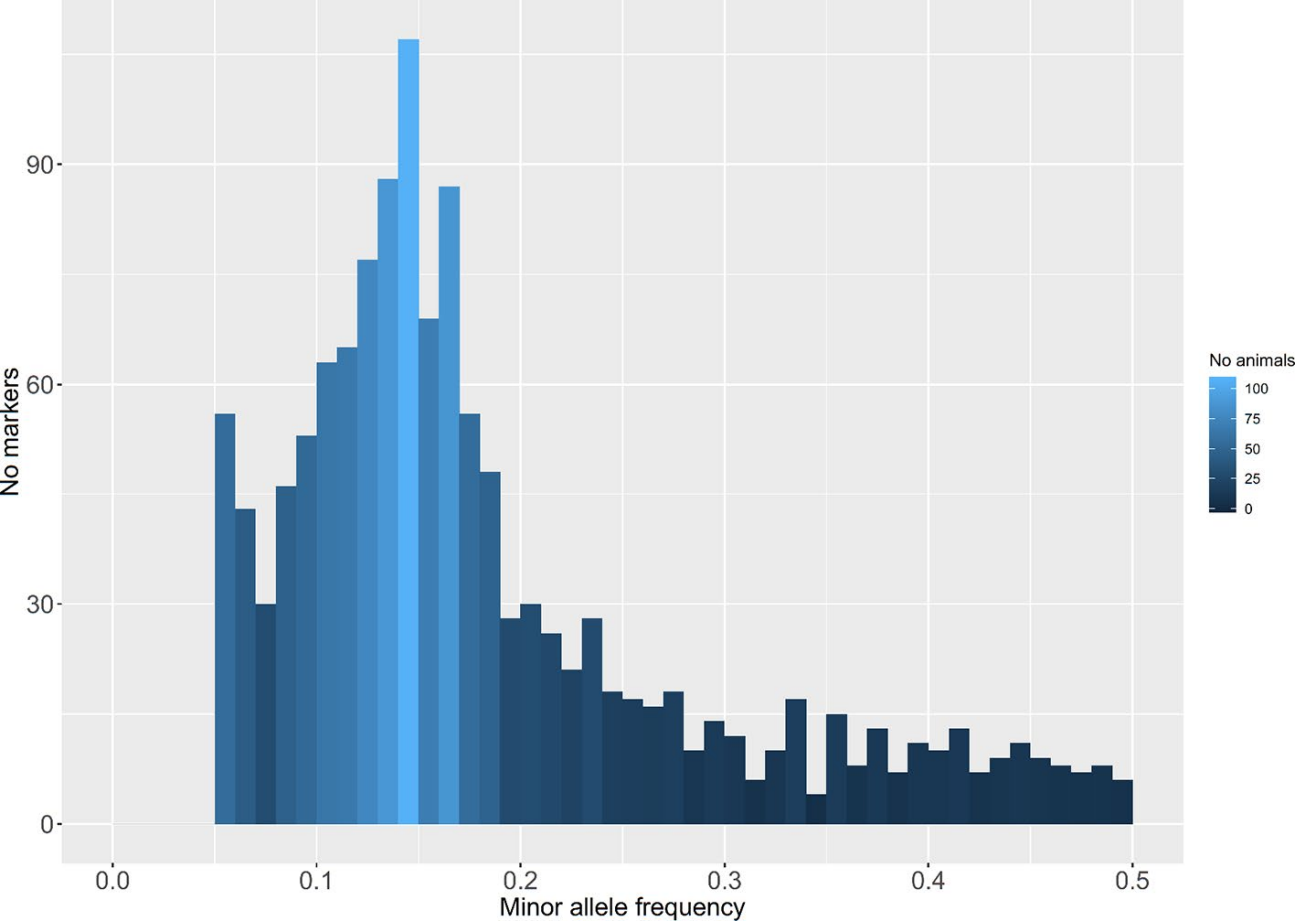
Distribution of minor allele frequencies of double-digest restriction site-associated DNA (ddRAD)–derived single nucleotide polymorphisms (SNPs) in seven populations of Nile tilapia.

A total of 169 million raw sequence reads (150 bp paired-end) were obtained. Approximately 140 million reads from 139 individual fish (one fish was removed due to sequencing failure) passed the aforementioned quality control (QC) filters. Alignment of these filtered reads to the Nile tilapia reference genome (Conte et al., 2017) resulted in a mapping rate of 94–97%. In total, 31,602 putative ddRAD loci corresponding to the restriction enzymes cutting sites were identified out of which 6,779 loci were polymorphic. Derived loci had a mean sequence coverage of 120X (SD = 60X). 3,821 polymorphic sites were removed due to missing values (>25%). In addition, 778 polymorphic loci were discarded due to low MAF values (<0.05). A total of 2,180 SNPs with a MAF above 0.05 across all samples (**Figure 2**) and found in more than 75% of the genotyped fish on each population were retained for downstream analysis. The mean MAF within populations ranged from 0.07 (Kunduchi) to 0.17 (TAFIRI).

### Genetic Similarity and Relationship Among Populations

The overall mean expected heterozygosity within populations was 0.132, while the observed heterozygosity was 0.081 (**Table 1**). Expected heterozygosity ranged from 0.057 in the FETA population to 0.214 in the Kunduchi population, while observed heterozygosity ranged from 0.057 in FETA to 0.113 in Ruhila (**Table 1**). Inbreeding coefficient (Fis) values ranged from low values in Lake Victoria (0.005), FETA (0.006), and Igunga (0.009) to relatively high values in Karanga (0.265), Ruhila (0.275), and Kunduchi (0.557).

**Table 1:** Summary of diversity parameters for the seven Nile tilapia populations.

Population	He	Ho	Fis
Ruhila	0.212	0.113	0.275
Karanga	0.213	0.104	0.265
TAFIRI	0.1	0.096	0.021
Kunduchi	0.214	0.067	0.557
Igunga	0.065	0.064	0.009
Lake Victoria	0.061	0.061	0.005
FETA	0.057	0.057	0.006

**Figure 3:**
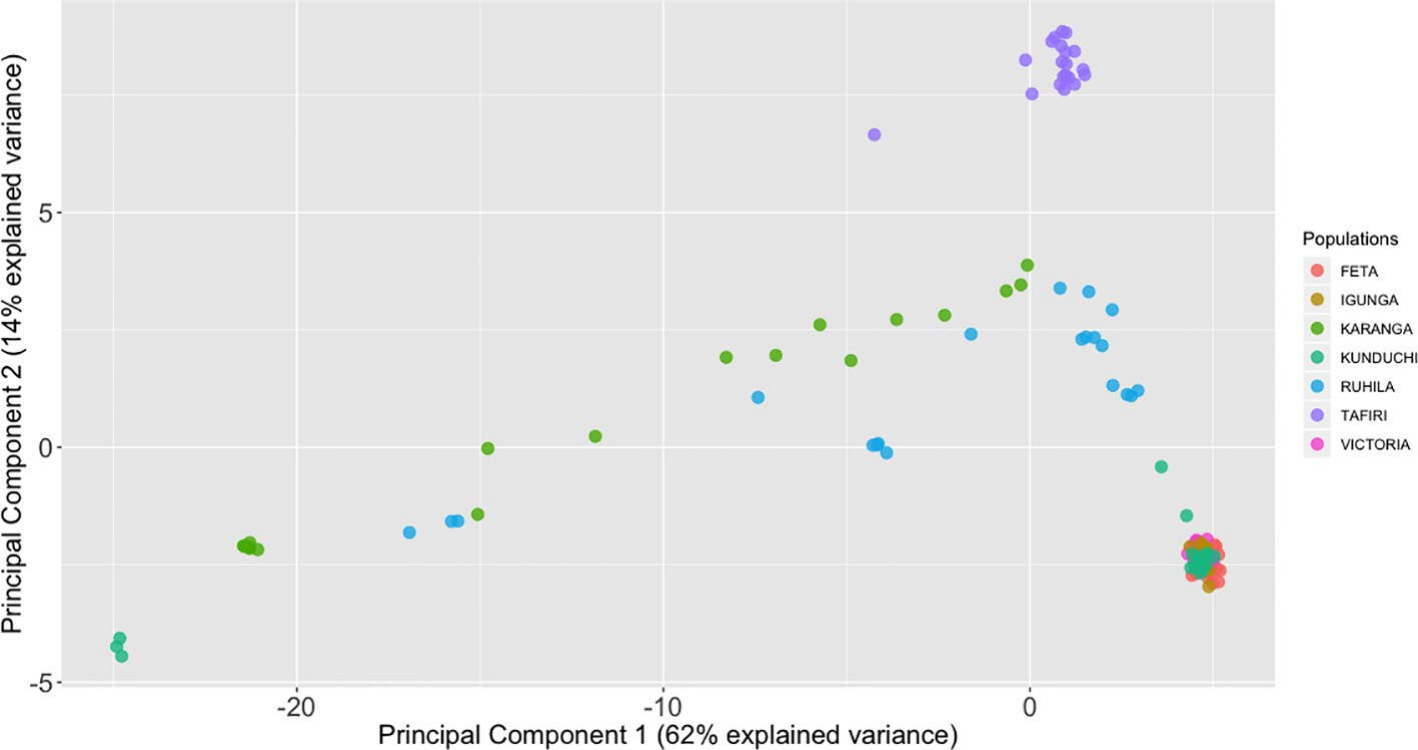
Principal components analysis (PCA) of the population for 139 fish individual fish based on 2,180 single-nucleotide polymorphisms (SNPs). The genetic relationships among individual fish as seen when plotting the first and second principal components (PCA1 and PCA2). Each individual is represented by one dot, with its symbol color corresponding to the assigned population.

Principal component analysis (PCA) was used to visualize individual relationships within and between populations. The first and second principal components accounted for 62% and 14% of the total variation, respectively. Individual fish from FETA, Lake Victoria, Igunga and most of the individual fish from Kunduchi formed a group of genetically similar animals (**Figure 3**). All TAFIRI fish formed a different group and were distinct from the other populations, except for one individual. Three individual fish from Kunduchi, one from TAFIRI, seven from Ruhila, and eight from Karanga did not group with the majority of animals from the same sampling locations.

The population pairwise F_ST_ values varied from 0.037 to 0.548 (**Table 2**). Lowest F_ST_ values were between Igunga and populations from the Lake Victoria and FETA. On the other hand, the highest F_ST_ values were between Karanga and the three most geographically distant populations, FETA, Lake Victoria and Igunga (F_ST_ = 0.548, 0.538, and 0.533 respectively). In addition, analysis of molecular variance (AMOVA) was used to detect within and among populations genetic variance components. AMOVA showed the highest levels of genetic variation within populations 67%, of the total variation, and 33% of variation was distributed among populations.

**Table 2:** Pairwise F_st_ with 95% confidence intervals (CI) among the seven population: TAFIRI, Ruhila, FETA, Lake Victoria, Karanga, Igunga and Kunduchi.

Population 1	Population 2	Lower CI	Upper CI	F_st_
TAFIRI	Ruhila	0.17890	0.20797	0.19322
TAFIRI	FETA	0.42571	0.47996	0.45256
TAFIRI	Victoria	0.40533	0.45925	0.43243
TAFIRI	Karanga	0.42979	0.45994	0.44498
TAFIRI	Igunga	0.39132	0.44652	0.41922
TAFIRI	Kunduchi	0.24015	0.27814	0.25967
Ruhila	FETA	0.24998	0.27900	0.26439
Ruhila	Victoria	0.23112	0.25824	0.24545
Ruhila	Karanga	0.19066	0.21102	0.20097
Ruhila	Igunga	0.22390	0.25150	0.23791
Ruhila	Kunduchi	0.06998	0.08676	0.07830
FETA	Victoria	0.08070	0.10897	0.09429
FETA	Karanga	0.53479	0.56045	0.54758
FETA	Igunga	0.03443	0.05096	0.04283
FETA	Kunduchi	0.10584	0.12052	0.11242
Victoria	Karanga	0.52577	0.55252	0.53849
Victoria	Igunga	0.02878	0.04490	0.03670
Victoria	Kunduchi	0.10591	0.11914	0.11226
Karanga	Igunga	0.51982	0.54576	0.53282
Karanga	Kunduchi	0.30078	0.32315	0.31192
Igunga	Kunduchi	0.08748	0.09959	0.09326

### Population Genetic Structure

**Figure 4:**
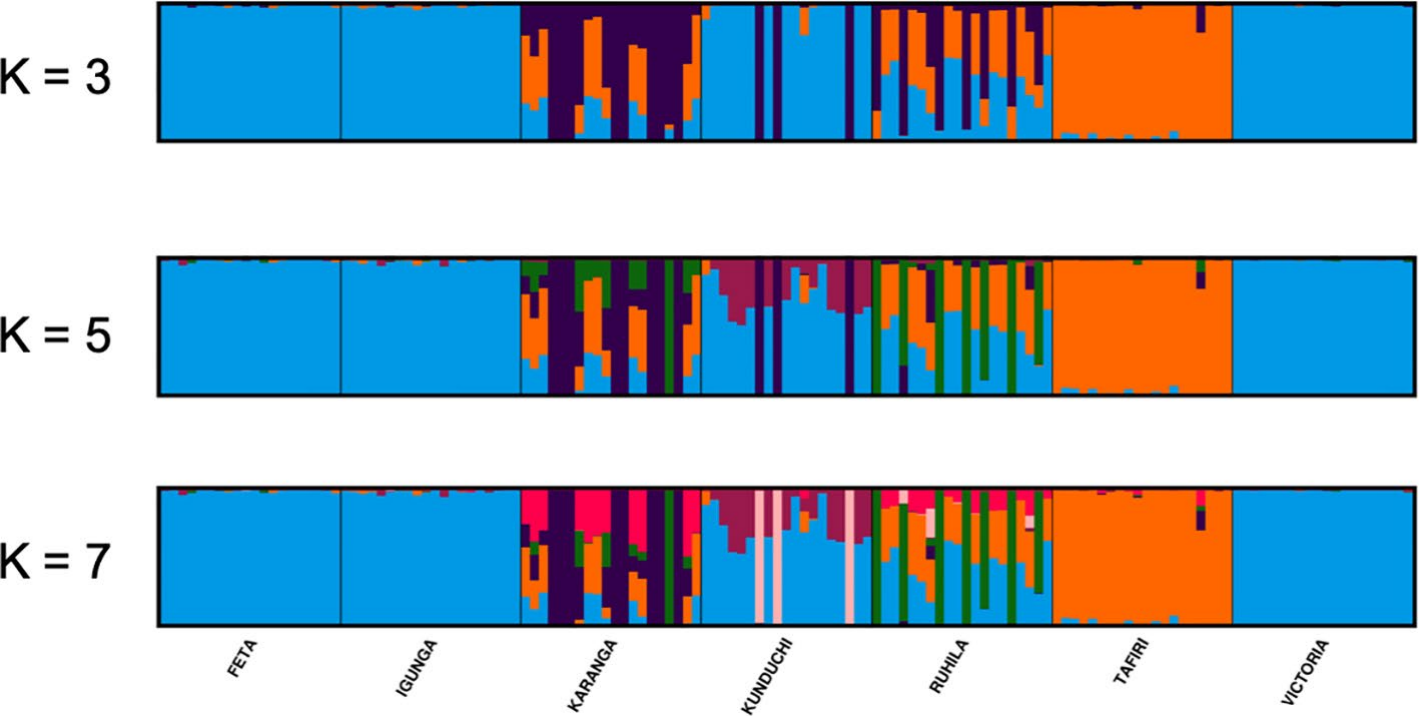
STRUCTURE analysis bar plots for K = 3, 5, and 7 (admixture model) showing population structure of different Nile tilapia sub-populations. Each vertical stripe represents an individual. Each color represents the proportion of membership with regard to the each assigned to seven genetic clusters. Same color in different individual fish indicates that they belong to the same cluster.

**Figure 5:**
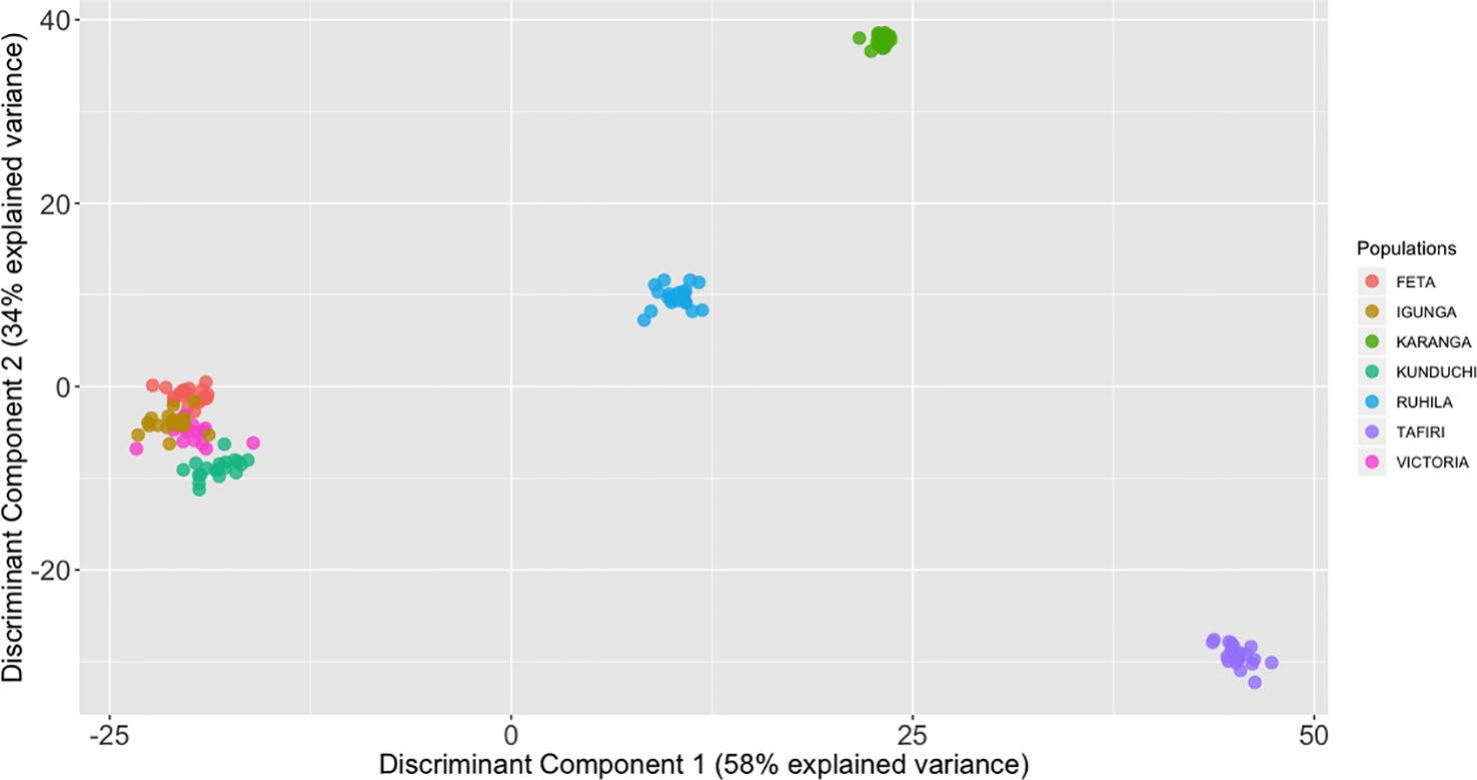
Discriminant analysis of principal components (DAPC) analysis with the *find.clusters* for 139 individual fish of the *O. niloticus* cultured in Tanzania. The axes represent the first two linear discriminants (LD). Squares represent groups and dots represent individual fish. Numbers represent the different populations identified by DAPC analysis.

The STRUCTURE analysis suggested that K = 7 was the most probable number of separate clusters for the studied Nile tilapia populations. Further, individual fish from FETA, Lake Victoria, Igunga and most of individual fish from Kunduchi (16 animals) appeared to share the same genetic cluster, while animals from TAFIRI formed a separate isolated cluster (**Figure 4**). Samples from the Karanga and Ruhila populations provided evidence of admixture. In addition, the existence of unique genetic clusters is suggested for both the Karanga and Ruhila populations. The aforementioned population structure was further validated in the DAPC analysis (**Figure 5**).

### Population Assignment and Diagnostic Single Nucleotide Polymorphisms

**Figure 6:**
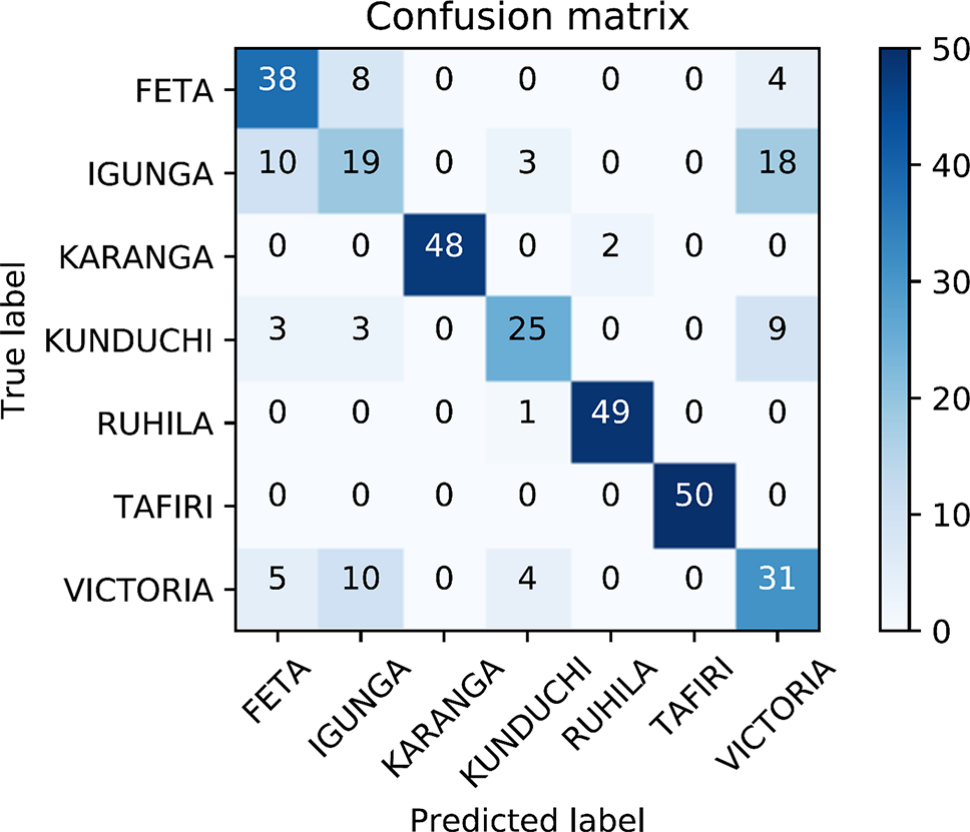
Confusion matrix for prediction efficiency of the single-nucleotide polymorphism (SNP) dataset using cross-validation. Four-fold cross-validation was performed where five randomly chosen animals on each population were considered of unknown origin. The entire procedure was repeated 10 times in order to minimize potential bias due to sample allocation in the training/test datasets. The diagonal contains the number of correct population assignments for the overall sum of the cross-validation scheme. Off-diagonals contain the number of erroneously population allocations for each particular case.

The identified SNP dataset was used for predicting the population of origin of putative unknown samples. An assignment rate of 77% was observed from the four-fold cross-validation analysis. The lowest correct allocation was obtained for samples from Lake Victoria, Kunduchi and Igunga (**Figure 6**). Mistakenly allocated samples were in all cases predicted as originating from either three populations (Lake Victoria, Kunduchi and Igunga). The aforementioned populations had the lowest genetic diversity values among them and formed a single cluster in the population structure analysis. In addition, DAPC analysis detected two SNPs with highest value for population identification. SNP-23095_6 and SNP-7137_40 had the highest population discriminatory value, indicating that they are the ones contributing most to cluster identification.

## Discussion

Understanding the patterns and extent of genetic divergence is essential both for efficient management of wild populations and for aquaculture activities. Many natural populations in Africa are under threat due to habitat destruction, overfishing and unregulated fish transfers (Eknath and Hulata, 2009). Furthermore, despite the value of Nile tilapia for the aquaculture sector in Tanzania limited research has been conducted regarding the genetic diversity of Nile tilapia populations in the country. The advent of ddRAD-seq and similar platforms have provided a cost effective and efficient technique for high resolution population genomic studies in many species (Peterson et al., 2012; Robledo et al., 2018). In this study, 2,180 SNP markers derived from ddRAD-seq were used to assess the genetic diversity and population structure of both locally cultured and wild *Oreochromis niloticus* strains in Tanzania.

From a farming perspective, evaluation of the genetic diversity among and within tested populations is crucial in order to ensure that the most diverse animals are chosen for selective breeding practices. Since Tanzania is a hot spot for tilapias, knowledge regarding genetic diversity will also be useful in appropriate management of wild populations. In addition, genetic variation is important for a population’s adaptation capacity towards changing environmental conditions (Fischer et al., 2017). Mireku et al. (2017) found higher genetic variation within populations than among populations in Nile tilapia populations from Lake Volta in Ghana. In this study AMOVA revealed the existence of higher genetic variation within populations than between populations. This could highlight that the usage of molecular markers (*e.g.* SNP data) would be of importance in future selective breeding practices as it would allow to utilize more efficiently the within population variance as opposed to traditional pedigree practices solely relying on the usage of passive integrated transponder tags. Nevertheless, as revealed by STRUCTURE analysis it should be taken into account that some populations contain unique genetic clusters not represented by “pure” populations.

Heterozygosity is a commonly used metric to compare the amount of genetic variation within different populations (Templeton and Read, 1994; Gu et al., 2014). Two different measures of heterozygosity are commonly used the observed and the expected heterozygosity. Gu et al. (2014) found that observed heterozygosity (Ho = 0.4483) in six *Oreochromis* populations in the primary rivers of Guangdong province were lower than the expected heterozygosity (He = 0.7097). On the contrary, Mireku et al. (2017) showed that observed heterozygosity (Ho = 0.526) of nine populations of *O. niloticus* in the Volta lake of Ghana was slightly higher than the expected heterozygosity (He = 0.459). In addition, Hassanien and Gilbey (2005) reported that the average of expected and observed heterozygosity were higher in *O. niloticus* populations from river Nile (He = 0.884 and Ho = 0.815) than from Delta lake populations (He = 0.846 and Ho = 0.533). In our study the overall observed heterozygosity (Ho = Ho = 0.081) was lower than the expected heterozygosity (He = 0.132) for most tested populations. Even though our study used SNP markers opposed to the aforementioned studies where microsatellites were primarily used the heterozygosity values are low compared to ddRAD studies in other fish species ranging between 0.18 and 0.25 (Saenz‐Agudelo et al., 2015). A possible explanation could be due to the low MAF in our SNP dataset. In particular, over 80% of the utilized SNPs had MAF below 0.2. In addition, our results could be partly explained due to the occurrence of non-random mating. Furthermore, the low heterozygosity levels could be explained by the Wahlund effect (Wahlund, 1928) where observed heterozygosity is reduced as populations diverge. We need also to acknowledge the potential influence of the relatively small to moderate sample size for each population (20 animals per population). Nevertheless, estimates of heterozygosity from empirical data are relatively insensitive to sample size (Allendorf and Luikart, 2007).

Populations from FETA, Lake Victoria and Igunga showed the same level of expected and observed heterozygosity suggesting that random mating potentially occurred (Templeton and Read, 1994). This is further supported by the low values of inbreeding coefficients (Fis) in the populations of Igunga, FETA and Lake Victoria. High positive Fis values indicate the existence of non-random mating or population subdivision. An additional explanation for the above could be also due to the existence of null alleles. Nevertheless, since the observed excess of homozygotes appears to occur on a population level rather than locus specific we would not expect the observed excess of homozygotes to be due to the existence of null alleles. The higher diversity in Kunduchi, Karanga and Ruhila populations on the other hand may be due to both the existence of non-random mating and due to a higher degree of admixture as revealed by the STRUCTURE analysis.

Genetic differentiation among populations is further affected by migration, mutation, drift, habitat heterogeneity and selection (Holsinger and Weir, 2009). Thus the actual levels of differentiation will be a balance between the homogenizing effects of gene flow due to the former and the disruptive effects of the latter (Allendorf and Luikart, 2007). Low-moderate levels of differentiation (Fst = 0.074) have been reported between the wild Nile tilapia from Lake Volta and the improved Akosombo strains in Ghana (Mireku et al., 2017). Also low degree of differentiation (Fst = 0.0297) was found between Nile tilapia populations from rivers of the Guangdong province in China. In our study genetic differentiation among FETA, Igunga and Lake Victoria populations was particularly low (F_ST_ values: 0.043 and 0.037 respectively). The similarity among these three populations is probably due to their origin from the same region of Lake Victoria (personal communication with fish farmers). According to our records the parents of the genotyped fish from FETA and Igunga also originated from Lake Victoria. Therefore, it is likely that these populations are genetically similar to each other and share the same genetic background. Moreover, the assignment of FETA, Lake Victoria and Igunga in the same cluster according to both STUCTURE and DAPC analysis provides further support for the aforementioned hypothesis. Nevertheless, in the case of TAFIRI a different trend was observed despite originating from the same location. The high F_ST_ values between TAFIRI and other populations (FETA, Igunga, and Lake Victoria) indicate high isolation between them. Interestingly, the TAFIRI population was composed of animals being in captivity for 4–6^th^ generations (personal communication with a fish farmer) and this could be a reason for its genetic uniqueness. Furthermore, we observed strong genetic differentiation between Karanga and the three closely related populations of FETA, Igunga, and Lake Victoria (F_ST_ = 0.548 0.538 0.533 respectively). The differences could be the result of geographical isolation which probably has acted as a barrier to gene flow between those populations, leading to the suggested genetic structure that the STRUCTURE analysis revealed. Nevertheless, gene flow is expected to have occurred among the admixed populations (Karanga, Ruhila, Kunduchi) and expected “pure” populations of Lake Victoria and TAFIRI. Since reproductive viable hybrids in tilapias are common (Wohlfarth and Hulata, 1983), the observed admixture in Karanga population could alternatively indicate that some animals could have been mistakenly described as pure Nile tilapia. Lowe et al. (2000), reported that it is particularly difficult to identify hybrids between the species based on morphology.

Multiple approaches using both multivariate analysis (PCA, DAPC) and Bayesian clustering algorithms (STRUCTURE) were used in the current study for deriving the underlying genetic structure among the sampled populations. PCA offers considerable advantages, since it can be applied in large datasets at a minimal computational cost compared to Bayesian approaches. In general terms, PCA aims to summarize the total variation between individuals in a reduced dimension. Nevertheless, the above approach does not necessarily provide optimal resolution for distinguishing between different groups. As such, approaches like DAPC have been shown to be particularly advantageous, since they retain the computational advantages of PCA, while at the same time offer higher resolution for detecting groups of individuals with common genetic background (Jombart et al., 2010). Animals from Kunduchi, Lake Victoria, FETA and Igunga clustered together. In contrast, fish from the TAFIRI population showed greater genetic differentiation appearing separated from the other populations. Interestingly, animals from TAFIRI did not group together with FETA, Igunga and Lake Victoria despite the fact that all the populations were sampled from the same region. Differences in allele frequencies between TAFIRI and other populations might be due to the use of relatively few founder stocks and possibly unforeseen reproductive bottlenecks. Other reasons could be due to founder effects and genetic drift because of small number of parents used for breeding.

Admixture analysis further supported that FETA, Lake Victoria, and Igunga together with animals from Kunduchi shared similar genetic background. On the other hand, high admixture levels were inferred in the Karanga, Ruhila, and Kunduchi populations. In the Ruhila population admixture with the population from Lake Victoria and TAFIRI was suggested. Moreover, a similar result was obtained for the Karanga population, while in the case of Kunduchi admixed fish shared genome variation with populations of FETA, Lake Victoria, and Igunga. The speculated uncontrolled movement of fish between different locations in- and outside Tanzania, maybe from Kenya or Thailand, could be an explanation for the suggested population admixture. Nevertheless, it needs to be stressed that both Ruhila and Kunduchi appear to contain animals of a distinct genetic background.

It should be stressed that the Ruhila aquaculture development center located in the southern part of Tanzania, stocked fish from Kingolwira aquaculture center in Morogoro in 2011. The Kingolwira aquaculture center obtained their broodstock from Lake Victoria. Native species to Lake Victoria are *O. esculentus* and *O. variabilis* while *O. leucostictus* and *O. niloticus* were introduced in the lake in 1950s (Bradbeer et al., 2018). Furthermore, Shechonge et al. (2019) found evidence of introduced *Oreochromis leucostictus* males from Ruhila government pond in Songea and also reported that fish farmers misidentified *O. leucostictus* as *O. niloticus*. Additionally, in the case of Karanga population native *Oreochromis* species found in Pangani basin including Lake Jipe are *O. jipe*, *O. pangani* and introduced *O. niloticus* and *O. esculentus* (Shechonge et al., 2019). As such species available at Karanga station are *O. pangani*, *O. niloticus*, *O. jipe* and probably hybrids of three species. This could explain the high admixture level in Karanga populations compared to other populations. Overall, the high suggested admixture level for Ruhila and Karanga populations could be due to potential mislabeled samples that were wrongly classified as Nile tilapia.

The current study attempted to investigate the efficiency of the SNP dataset for population discrimination purposes of potentially unknown origin samples using a cross-validation scheme. The ability to predict the population of origin is most valuable both for fish farming practices and for conservation purposes of wild populations. Separating the dataset in a training and a validation set was applied in order to minimize overfitting, a commonly encountered situation especially in models with a considerable larger size of predictors (SNP data) than samples (genotyped fish). Model overfitting in our case could mistakenly lead to the conclusion that the SNP dataset would be highly efficient in deciphering the most probable population of origin of unknown samples. Overall 77% of tested individual fish were correctly allocated to population of origin using the SNP data. Most of the erroneous assignments originated from the three closely related populations for which our information suggests that all three originate from Lake Victoria. Further, a low number of correctly assigned individual fish were obtained in the Kunduchi population. As suggested both by STRUCTURE and DAPC high level of admixture is suggested for the Kunduchi population. Taking the above into account successful assignment to population of origin exceeded 92%. Nevertheless, it needs to be acknowledged that for the conducted analysis to be most efficient the population information of the training dataset should be highly accurate. The expected unregulated transferring of fish in Tanzania coupled with the inherent difficulty of species discrimination among tilapias using phenotypic criteria and the most common hybridization between tilapia species resulting to reproductive viable offspring could suggest that potentially mislabeled samples have been included.

Overall, the obtained results from our study indicate that the genetic diversity and structure of Nile tilapia populations cultured in Tanzania can be explained by their life history and geographical distribution. The results also revealed greater genetic diversity within than among populations. The close clustering of Igunga, FETA and Lake Victoria populations and distinct separation of TAFIRI, suggests that these could be pure populations without admixture. The above should be taken into consideration in future wild populations conservation practices. Moreover, the gained information regarding population structure among the tested tilapia populations is important for characterizing genetic similarities and relationships of cultured lines in Tanzania. Understanding how genetic variation is distributed within and among populations will facilitate the formation of a base population and will allow breeders to design crossings between the aforementioned populations in order to maximize the genetic diversity for selective breeding purposes. Therefore, the results from this study could be used as a guide for future breeding programs and genetic improvement of local Nile tilapia in Tanzania, which may ultimately form an exemplar for the development of local tilapia species and breeds for aquaculture in African countries. Finally, using SNP data to infer the population of origin is of great importance not only for estimating genetic diversity but also in wild population conservation practices. There are unique tilapia species in Tanzania that must be protected and preserved. In addition, the SNP dataset developed can also be valuable for traceability purposes especially with regards to wild populations inhabiting nature protected reservoirs.

## Data Availability Statement

The aligned reads in the format of bam files were deposited in the National Centre for Biotechnology Information (NCBI) repository under project ID PRJNA518067. The accession numbers of samples analyzed in this study are given in **File S1**.

## Author Contributions

RK and FP carried out DNA extraction. CP and RH performed ddRAD library preparation and sequencing. DK, MM, and RK framed the study and contributed to designing the experiments. MM and AM provided valuable suggestions to the manuscript. RK and CP performed the statistical and genetic analyses. RK wrote the manuscript. DK and CP revised the manuscript. All authors approved the final draft of the manuscript.

## Conflict of Interest

The authors declare that the research was conducted in the absence of personal or financial relationships that could be construed as a potential conflict of interest
